# Supplementary material for: A porcine model of Fanconi anemia
Source: PLoS One. 2025 Oct 31;20(10):e0335854. doi: 10.1371/journal.pone.0335854 (PMC12578174; doi:10.1371/journal.pone.0335854)
Supplement: S1 Table — Outcome of initial targeting is shown. (DOCX) [file pone.0335854.s001.docx]

| **ID** | **Allele 1** | **Allele 2** |
| --- | --- | --- |
| 115-1 | WT | -3 |
| 115-2 | WT | WT |
| 115-3 | -3 | +1/-3 |
| 115-4 | WT | -2 |
| 115-5 | WT | WT |
| 115-6 | WT | WT |
| 115-7 | WT | +2 |
| 115-8 | WT | +7 |
| 115-9 | WT | WT |
| 115-10 | WT | -8 |

**Table S1. FANCA exon 4 targeted FANCA pigs**
