# Supplementary material for: A porcine model of Fanconi anemia
Source: PLoS One. 2025 Oct 31;20(10):e0335854. doi: 10.1371/journal.pone.0335854 (PMC12578174; doi:10.1371/journal.pone.0335854)
Supplement: S2 Table — Offspring from IVF treated sow 126 and 127 are shown. *Could not identify mutations through sequencing. (DOCX) [file pone.0335854.s002.docx]

| **ID** | **Allele 1** | **Allele 2** |
| --- | --- | --- |
| 126-1 | WT | WT |
| 126-2 | WT | -207 |
| 126-3 | +146 ex31 / -1 ex32 | -3 |
| 126-4 | WT | WT |
| 126-5 | WT | WT |
| 126-6 | -215 | WT |
| 126-7 | WT | -1 ex31 / +1 ex32 |
| 127-1 | WT | WT |
| 127-2 | -230 | WT |
| 127-3 | WT | WT |
| 127-4 | WT | WT |
| 127-5 | WT | WT |
| 127-6 | WT | WT |
| 127-7 | Complex* | Complex* |

**Table S2. FANCD2 Exon 31/32 targeted F0 pig generation.**

Offspring from IVF treated sow 126 and 127 are shown.

*****Could not identify mutations through sequencing.
