# Supplementary material for: A porcine model of Fanconi anemia
Source: PLoS One. 2025 Oct 31;20(10):e0335854. doi: 10.1371/journal.pone.0335854 (PMC12578174; doi:10.1371/journal.pone.0335854)
Supplement: S3 Table — * 126−2 has an additional mosaic −3 mutation in exon 32. †126−3 has an additional mosaic −86 mutation in exon 31 and +226 mutation in exon 32. Fetal harvest at day 35 of gestation. Fetal harvest at day 26 of gestation. (DOCX) [file pone.0335854.s003.docx]

| **ID** | **Sex** | **Allele 1** | **Allele 2** |  | **ID** | **Sex** | **Allele 1** | **Allele 2** | **Litter ID** |
| --- | --- | --- | --- | --- | --- | --- | --- | --- | --- |
| 126-2* | Gilt | WT | -207 | X | 126-6 | Boar | -215 | WT | 46 |
| 126-3† | Gilt | +146 ex 31 / -1 ex 32 | -3 | X | 126-6 | Boar | -215 | WT | 59 |
| 127-2 | Gilt | -230 | WT | X | 126-7 | Boar | WT | -1 ex 31 / +1 ex 32 | 23 |
| 126-3 | Gilt | +146 ex 31 / -1 ex 32 | -3 | X | 126-6 | Boar | -215 | WT | 86 |
| **ID** | **Sex** | **Allele 1** | **Allele 2** |  | **ID** | **Sex** | **Allele 1** | **Allele 2** | **Fetal ID** |
| 126-3 | Gilt | +146 ex 31 / -1 ex 32 | -3 |  | 126-6 | Boar | -215 | WT | 82‡ |
| 59-3 | Gilt | +146 ex 31 / -1 ex 32 | WT |  | 126-6 | Boar | -215 | WT | X§ |

**Table S3. FANCD2 exon 31/32 targeted breeding pairs.**

* 126-2 has an additional mosaic -3 mutation in exon 32.

†126-3 has an additional mosaic -86 mutation in exon 31 and +226 mutation in exon 32.

‡ Fetal harvest at day 35 of gestation.

§ Fetal harvest at day 26 of gestation.
