# Supplementary material for: A porcine model of Fanconi anemia
Source: PLoS One. 2025 Oct 31;20(10):e0335854. doi: 10.1371/journal.pone.0335854 (PMC12578174; doi:10.1371/journal.pone.0335854)
Supplement: S4 Table — Litter 46–126−2 x 126−6; Litter 59–126−3 x 126−6; Litter 23–127−2 x 126−7; Litter 86–126−3 x 126−6. (DOCX) [file pone.0335854.s004.docx]

| **ID** | **Allele 1** | **Allele 2** |
| --- | --- | --- |
| 46-1 | -3 | WT |
| 46-2 | -3 | WT |
| 46-3 | -3 | WT |
| 46-4 | -3 | -215 |
| 59-1 | WT or -3 | -215 |
| 59-2 | WT or -3 | -215 |
| 59-3 | +146 ex31 / -1 ex32 | WT |
| 59-4 | -3 | WT or -3 |
| 59-5 | WT | -215 |
| 59-6 | -146 | WT |
| 59-7 | WT | WT or -3 |
| 59-8 | +146 ex31 / -1 ex32 | -3 |
| 59-9 | WT or -3 | -215 |
| 23-1 | WT | WT |
| 23-2 | -1 ex31 / +1 ex32 | WT |
| 23-3 | WT | WT |
| 23-4 | WT | WT |
| 23-5 | -230 | WT |
| 23-6 | -230 | WT |
| 23-7 | -230 | WT |
| 86-1 | +146 ex31 / -1 ex32 | WT |
| 86-2 | -3 | -215 |
| 86-3 | +146 ex31 / -1 ex32 | WT |
| 86-4 | -3 | WT |
| 86-5 | -3 | -215 |
| 86-6 | -3 | WT |
| 86-7 | -3 | -215 |
| 86-8 | -3 | -215 |

**Table S4. FANCD2 F1 generation pig litters.**

*Litter 46 – 126-2 x 126-6; Litter 59 – 126-3 x 126-6; Litter 23 – 127-2 x 126-7; Litter 86 – 126-3 x 126-6*
