# Supplementary material for: A porcine model of Fanconi anemia
Source: PLoS One. 2025 Oct 31;20(10):e0335854. doi: 10.1371/journal.pone.0335854 (PMC12578174; doi:10.1371/journal.pone.0335854)
Supplement: S5 Table — Pig fetuses harvested in early gestation are shown. (DOCX) [file pone.0335854.s005.docx]

| **ID** | **Allele 1** | **Allele 2** |
| --- | --- | --- |
| 82-1 | +146 ex31 / -1 ex32 | WT |
| 82-2 | -3 | -215 |
| 82-3 | +146 ex31 / -1 ex32 | WT |
| 82-4 | -3 | WT |
| 82-5 | -3 | -215 |
| 82-6 | -3 | WT |
| 82-7 | -3 | -215 |
| 82-8 | -3 | -215 |
| X-1 | WT or -3 | -215 |
| X-2 | WT or -3 | -215 |
| X-3 | WT or -3 | -215 |
| X-4 | WT or -3 | -215 |
| X-5 | WT or -3 | -215 |
| X-6 | WT or -3 | +146 ex31 / -1 ex32 |
| X-7 | WT or -3 | +146 ex31 / -1 ex32 |
| X-8 | WT or -3 | +146 ex31 / -1 ex32 |
| X-9 | WT or -3 | WT |
| X-10 | WT or -3 | -215 |
| X-11 | WT or -3 | -215 |

**Table S5. FANCD2 exon 31/32 F1 generation fetal genotypes.**

Pig fetuses harvested in early gestation are shown.
