# Supplementary material for: A porcine model of Fanconi anemia
Source: PLoS One. 2025 Oct 31;20(10):e0335854. doi: 10.1371/journal.pone.0335854 (PMC12578174; doi:10.1371/journal.pone.0335854)
Supplement: Attachment — Submitted filename: Response to Reviewers.docx [file pone.0335854.s006.docx]

**Responses to Reviewers are in italics and underlined**

**Comments to the Author**

2. Has the statistical analysis been performed appropriately and rigorously?

Reviewer #1: No. *The information clearly describing the experimental design and the statistical analysis for Figure 6B has now been included*.

Reviewer #2: Yes

5. Review Comments to the Author

Reviewer #1:

In Figure 6B, an improper statistical test is performed. Even though it is not outlined what statistical test was used, the compared values are not independent, but multiple resampling of the same animal over time. *The information we provided for this assay was incomplete. We have revised Figure 6 B, the Methods section and the Figure legend to address this important issue*.

Please indicate whether the founder homozygous FANCA animals had any unusual birth defects such as the extra digit. *The biallelic animal (pig 67-2) had an extra dew claw on both front limbs and this is indicated in the text and shown in Figure 3*.

In the discussion it is noted that “mouse models of FA do not show skeletal defects” however this is incorrect. See 10.1016/j.semcdb.2020.11.010 for a discussion of the various examples that are published. *We agree that some mouse models show a variety of developmental defects in bone as discussed in the comprehensive review by Masson and colleagues. The text has been modified accordingly*.

The discussion should also more explicitly reference some of the potential clinical trials utilizing gene therapy and gene editing, for which this animal would be useful. *We have included some of the potential uses of this FA pig model that may have clinical relevance in the discussion section*.

Reviewer #2: …. several formatting and figure presentation issues should be addressed to improve clarity and readability. *See below*.

Major Issues:

- It is very interesting to observe that FANCD2 knock-out mice are lethal. However, it would be interesting to show if the depletion of FANCD2 in normal porcine fibroblasts presents typical cellular defects of Fanconi anemia as human cases. *FAND2 targeted pig fibroblasts showing the anticipated increased sensitivity to DEB in a colony forming assay is shown in Figure 4*.

Minor Issues:

- Figure Legends and Content Alignment: The presentation of Figure 1A is unclear. In the Results section, the authors begin by validating exon 4 targeting in wild-type porcine fibroblasts. However, Figure 1A does not indicate the specific cell type used in this experiment. Clarification in the figure legend and labeling would enhance interpretability.
- Figure Ordering: The order of figures appears disorganized. For example, the appearance of Figure 1A, then Figure 2A, followed by Figure 1B, disrupts logical flow. Figures should be reorganized to follow the sequence of experiments as presented in the text.
- Figure Quality: The resolution of several figures, particularly Figure 5, is suboptimal. High-resolution versions should be provided to allow proper evaluation of the data.
- Figure Consistency: There are inconsistencies in figure sizing and formatting. Some figures are disproportionately large compared to others. A standardized format should be adopted across all figures to maintain a cohesive presentation.

*Apparently 2 versions of the figures were sequentially uploaded. The first version had the figures out of order and there were resolution issues with several figures. This has been corrected in the revised manuscript with NAAS approved figures*.

*While revising your submission, please upload your figure files to the Preflight Analysis and Conversion Engine (PACE) digital diagnostic tool, <https://pacev2.apexcovantage.com/>. PACE helps ensure that figures meet PLOS requirements. *Done*
